# Clinical Implication of Brain Metastases En-Bloc Resection: Surgical Technique Description and Literature Review

**DOI:** 10.3390/jpm14111110

**Published:** 2024-11-19

**Authors:** Roberto Altieri, Sergio Corvino, Giuseppe La Rocca, Fabio Cofano, Antonio Melcarne, Diego Garbossa, Manlio Barbarisi

**Affiliations:** 1 Multidisciplinary Department of Medical-Surgical and Dental Specialties, University of Campania “Luigi Vanvitelli”, 80131 Naples, Italy; manlio.barbarisi@unicampania.it; 2 Department of Neurosciences, Reproductive and Odontostomatological Sciences, Division of Neurosurgery, University of Naples “Federico II”, 80131 Naples, Italy; sercorvino@gmail.com; 3 Institute of Neurosurgery, A. Gemelli University Polyclinic, IRCCS and Foundation, Sacred Heart Catholic University, 20123 Rome, Italy; giuseppe.larocca@policlinicogemelli.it; 4 Neurosurgery, Department of Neuroscience, University of Turin, Via Cherasco 15, 10126 Turin, Italy; fabio.cofano@unito.it (F.C.); anmelcarne@gmail.com (A.M.); dgarbossa@gmail.com (D.G.)

**Keywords:** brain metastases, en-bloc resection, piecemeal resection, leptomeningeal dissemination, recurrences

## Abstract

**Background:** The role of brain metastases (BM) surgery is of paramount importance for patients’ progression-free and overall survival. “En-bloc” and “piecemeal” resection represent the main surgical techniques. Although en-bloc resection remains the best surgical option, it is not widely adopted or feasible as the first choice. We describe our point of view about the en-bloc surgical technique with an illustrative case and discuss its indications with pros and cons through a comprehensive literature review. **Materials and methods:** A Medline search up to December 2023 in the Embase and PubMed online electronic databases was made and PRISMA statement was followed. An illustrative case of “en-bloc” resection from our surgical series was also added as a technical note. **Results:** We describe tips and tricks of our surgical technique and added a surgical video from our series. The literature review disclosed 19 studies. Resulting data suggested that “en-bloc” resection, when feasible, provides lesser risk of leptomeningeal dissemination, local recurrence rates, intraoperative bleeding occurrence and perioperative complications; in addition, it preserves the normal anatomy. **Conclusions:** En-bloc resection is the gold standard technique for surgical treatment of brain metastases especially for patients with superficial lesions that are small in size and far from eloquent areas.

## 1. Introduction

The en-bloc resection technique is a mainstay of oncological surgery in order to promote radicality and improve patient prognosis [1,2]. Generally, the en-bloc resection technique requires a clear and distinct interface between tumor and peritumoral tissue. Neurosurgeons pay less attention to performing this surgical practice compared to other surgical specialties. This can be explained by the attitude to preserve healthy brain tissue and from the experience of glioma surgery where there are no defined tumoral margins [3,4].

For this reason, also in brain metastases (BM) surgery, many neurosurgeons prefer the piecemeal resection and the usage of ultrasonic aspirator. The main reason for this surgical choice is to avoid the direct brain manipulation, reduce tumor volume and facilitate its removal also through minimally invasive craniotomy. However, the literature evidence suggests that intralesional debulking could cause tumoral cells to spread with risks of seeding and iatrogenic neoplastic meningitis [5,6]. Moreover, intralesional debulking could determine a diffuse bleeding with the necessity of a tedious use of bipolar coagulation with the risk of postoperative neurological deficits for vascular damage [7].

The aim of the present study was to describe the metastases en-bloc resection technique through an illustrative case and a comprehensive literature review. We discuss the main indications, tips and tricks and pros and cons of this surgical method of BM resection.

## 2. Materials and Methods

We discuss the technical note of the en-bloc resection performed on a young patient affected by single left frontal breast metastases. The consent of our patient was not required since her data were sufficiently anonymized.

**Figure 1:**
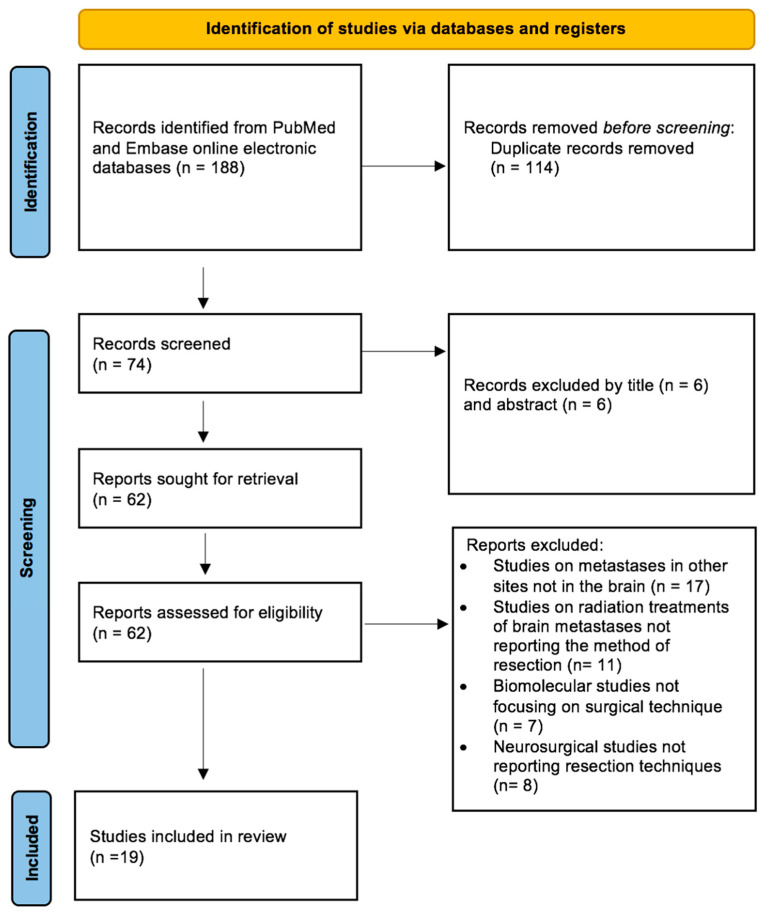
PRISMA (Preferred Reporting Items for Systematic Reviews and Meta-Analyses) guidelines. Systematic review of the literature to identify studies in the online MEDLINE (PubMed) and EMBASE reporting patients with brain metastases underwent surgery through en-bloc or piecemeal resection.

A systematic review of the literature was conducted in accordance with the PRISMA (Preferred Reporting Items for Systematic Reviews and Meta-Analyses) guidelines to identify studies in the online electronic EMBASE and PubMed databases reporting the resection techniques—en-bloc and piecemeal—of brain metastases (Figure 1). This study is not registered in any database.

The search strategy was designed for the identification of English-language articles published up to December 2023 using the following relevant keywords, alone or in combination: (((((((((brain metastases) OR (brain metastasis)) OR (cns metastasis)) OR (cns metastases)) AND (en bloc resection)) AND (en-bloc resection)) AND (piecemeal resection)) AND (surgical resection)) AND (resection technique)). Results were analyzed and processed with the ZOTERO reference manager. All papers written entirely in languages other than English were excluded. Papers discussing different topics (metastases involving other organs or brain metastases analyzed for reasons other than type of surgical technique) were excluded. Papers without full text were excluded. Time or publication status restrictions were not applied. Additional studies were added based on a review of bibliographies of the identified papers.

Inclusion criteria encompassed surgical series, reviews, case-control series in English language, as well as papers written in other languages, but including the abstract in English, involving patients with BM who underwent surgery, reporting the technique of resection adopted and the related risk of leptomeningeal dissemination and tumor recurrence.

## 3. Results

### 3.1. Technical Note

**Figure 2:**
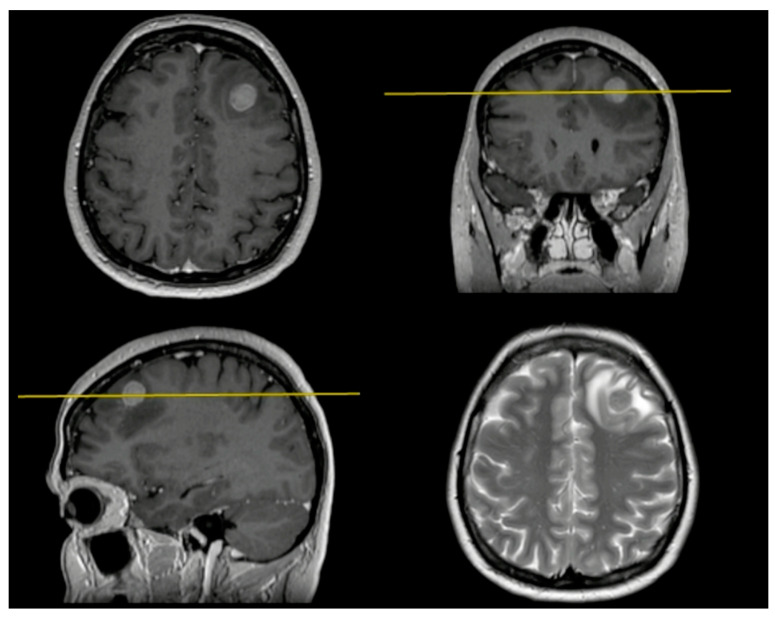
Preoperative MRI shows a BM located under the left middle frontal gyrus.

In this case example, we describe the en-bloc resection technique of BM. We present a 47 y.o. female patient with a previous history of breast cancer treated 1 year before. The neuroradiological findings showed a single metastasis located into the left frontal lobe (Figure 2). No more metastases were found after a total body CT scan.

The tumor was anatomically located in the withe-gray matter boundary under the middle frontal gyrus. The patient was placed in supine position with the head slight rotated on the left and fixed with a Mayfield clamp.

**Figure 3:**
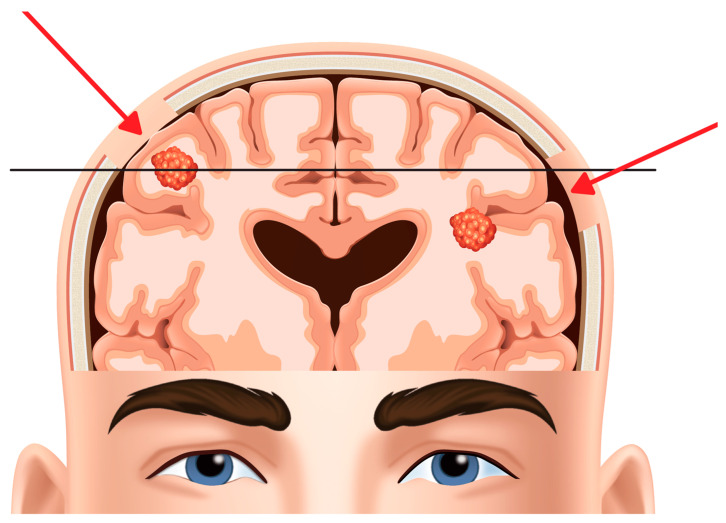
Figure shows the size of craniotomy centered on the target in order to manage the metastasis trough the trans-cortical or trans-sulcal approach based on the anatomical location of the tumor.

With the aid of image guidance, we performed a linear incision and a craniotomy centered on the target. The size of the craniotomy should avoid the exposure of healthy brain but be enough large to manage the entire cortical projection of the larger metastasis diameter to allow brain mapping (if needed). Exposure of the brain surface corresponding to the larger tumor diameter offers the possibility to gently manipulate the tumor over its entire surface during subpial disconnection maneuvers. If the tumor is located under the cortical layer of the gyrus (like in this case) we prefer a direct corticectomy. Otherwise, if the metastases is located under a sulcus, we choose a microsurgical trans-sulcal approach [8] (Figure 3).

**Figure 4:**
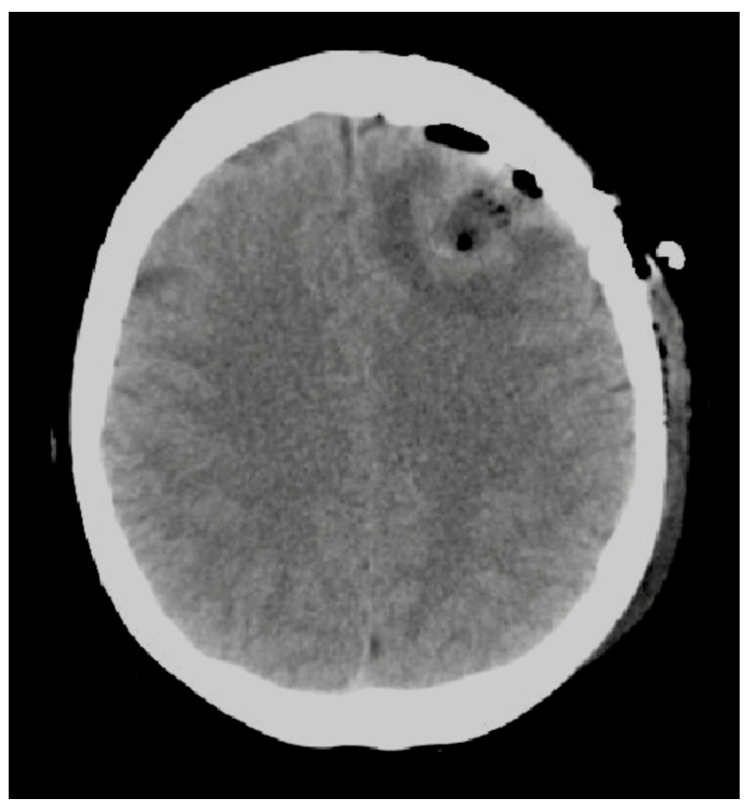
Shows a postoperative CT scan confirming the absence of surgical complications.

The dural opening was performed in order to show our target and cottonoids were used to protect the healthy cortex and avoid the epidural bleeding. After the identification of the tumor with the neuronavigation passive probe, the tumor resection started with a subpial disconnection technique leaving a thin shell of white matter around the lesion in order to reduce the spread of tumoral cells. The bipolar was used only as a tool to help the dissection maneuvers. Blunt and cold dissection with suction was used in order to avoid vascular and axonal thermic injuries. Moreover, we suggest the use, when needed, of low amperage bipolar coagulation to preserve normal anatomy and the cleavage plane which can be lost by the emulsification of the tissue induced by higher amperages. Furthermore, the gentle use of suction gives an important tactile feedback helping the surgeon to better identify the interface between the tumor and heathy tissue. In addition, the en-bloc resection by avoiding the progressive decompression via piecemeal debulking, reduces the brain shift effect and improves neuronavigation accuracy [7]. In this case, surgery via an en-bloc approach was obtained with minimal bleeds and without additional neurological deficit. The postoperative CT scan confirmed a complete resection of the tumor without complications (Figure 4) (see the Video S1).

The limitations of this approach are mainly related to the location, dimension and consistency of metastases. Large lesions in eloquent areas may require intralesional debulking to avoid traction on the surrounding brain. Cystic tumors can be more challenging to remove en-block but the technical difficulties can be overcome with careful dissection and, on some occasions, by using a neuronavigated needle aspiration of the fluid component. Some authors suggest the use of fibrin glue to replace the fluid component and facilitate the en-bloc excision [9,10].

### 3.2. Literature Review

A detailed and comprehensive systematic literature review disclosed 182 articles. After duplicate removal, all abstracts were evaluated, and each article of interest was marked for further review. The full text of the marked studies was screened by two authors independently and according to the inclusion criteria, 19 studies were included in this systematic review, as shown in Figure 1. Of the nineteen records identified, fourteen are clinical articles, one a metanalysis, three are reviews of the literature and one is a guideline (Table 1).

The “en-bloc” resection technique was first described by Salvati et al. in 1996 on a retrospective series of 19 patients affected by single cerebral metastases. In 10 cases the lesion was removed with circumferential disconnection named by authors as the “no internal touch technique”. They noted on these small series that survival was influenced by en-bloc resection and whole-brain irradiation [11].

Suki et al., in 2008, published a clinical article about the risk of leptomeningeal metastases after piecemeal resection of posterior fossa metastases. Authors retrospectively analyzed 260 patients and demonstrated that piecemeal tumor resection (137 cases) was strictly associated with a higher risk of leptomeningeal dissemination (LMD) than en-bloc tumor removal (123 cases) or sterotactic radiosurgery (SRS). Moreover, they assessed that there was no difference in the risk for the development of neoplastic meningitis between en-bloc resection and SRS [12].

One year later in a series of 827 patients with supratentorial metastases, these same authors confirmed the association between piecemeal resection and LMD risk. Moreover, their study provided evidence that the difference between the piecemeal and en-bloc resection techniques was particularly relevant in patients affected in melanoma metastases [13].

In 2010, Sawaya et Al underlined the impact of en-bloc resection on the survival rate in a series of 29 patients affected by intraventricular metastases [15].

Patel et al. investigated factors associated with local recurrence rates in BM. In a large series involving 570 patients they showed that the histological type did not influence the local recurrence rate; this risk is not increased by piecemeal resection and tumors with a volume higher than 9.7 cm^3^. The multivariate analysis in the same study has proven that small tumors removed with en-bloc resection had a lower risk of local recurrence [14].

Ahn JH et al. studied other anatomical and technical aspect associated with leptomeningeal spillage risk with development of meningeal metastasis. In a surgical series of 242 patients, they demonstrated that the risk of leptomeningeal dissemination was significantly higher in piecemeal resection group than in patients who underwent en-bloc resection (HR 4.08). Moreover, the authors underlined that the incidence of LMD was significantly higher in patients in whom an ultrasonic aspirator was used (HR 2.64). In addition, a tumor location close to the CSF increased the risk of LMD (HR 11.36). The authors concluded in their work that the risk of developing leptomeningeal disease was strongly associated with piecemeal resection, the use of the ultrasonic and distance between metastases and CSF [5].

Again, Patel et al., in a retrospective study involving a large sample (1033) of patients with single BM who underwent surgical resection, analyzed the impact of surgical methodology on the complication rate and functional outcome. Their results showed a significantly lesser overall complication rate for patients undergoing en-bloc resection than those undergoing piecemeal resection, with a similar but not significant trend towards major neurological complications. Therefore, the authors concluded that en-bloc resection of BM was not associated with higher postoperative complication rates, also for lesions in eloquent areas or large tumors, and it was at least as safe as the piecemeal resection method [16].

A radiation oncologist group evaluated 165 patients and did not find any relation between the resection technique and risk of leptomeningeal disease. Instead they correlated this risk with breast cancer histology [17].

Johnson et al. in their evaluation of the risk of LMD in an overall series of 465 patients with BM treated with surgery plus SRS to the surgical resection cavity compared with patients treated only with SRS found that the surgical technique was not associated with an increased risk of LMD [18].

Press et al. with the aim to investigate the association between hemorrhagic and cystic BM in 134 patients treated with surgical resection and SRS and the risk of LMD, noted that hemorrhagic and cystic features were independently associated with increased risk of postoperative LMD, whereas they did not find a correlation with the method of resection [19].

Demaerel et al. compared outcome in an overall series of 52 patients with a solitary supratentorial or infratentorial metastasis, with surgery performed as en-bloc resection in 87% of supratentorial cases and in 50% of infratentorial ones. They noted that carcinomatous meningitis was more frequently associated with the piecemeal resection technique [20].

Bruzzaniti et al. recently suggested on a retrospective surgical series of 88 patients that en-bloc tumor removal should be considered the gold standard treatment of BM especially in the presence of solitary BM with maximal diameter ≤2 cm but with high-grade oedema, since this treatment reduces the overall mass effect [21].

Byun et al. retrospectively analyzed 197 patients affected by BM from non-small cell lung cancer (NSCLC) and in multivariate analyses, they found that en-bloc resection was the only factor significantly correlated with a lower local/distant recurrence rate [22].

Kalyvas et al. analyzed the anatomical and surgical characteristics associated with pachymeningeal failure (PMF) in patients with BM after neurosurgical resection and adjuvant SRS and found that piecemeal resection was correlated to PMF in multivariate analysis [24].

In a recent meta-analysis including 13 studies and 2105 patients and aiming to systematically review and quantitatively assess risk factors for LMD after surgical resection for BM, Tewarie et al. found that subtotal or piecemeal resection was significantly associated to leptomeningeal disease recurrence [23].

Other authors wrote literature reviews based on the aforementioned papers that were also used to write the guideline of Congress of Neurological Surgeons suggesting (with recommendation level 3) that en-bloc resection should be preferred to decrease the risk of postoperative LMD when resecting single BM [9,25,26,27].

All these data are summarized in Table 1.

## 4. Discussion

BM from solid extracranial neoplasms are the most frequent intracranial tumors, with an incidence up to 10 times higher than primary malignant brain tumors and which is steadily growing due to their early detection, ageing population, advances in neuroimaging and in the treatment of the systemic disease [28]. They are diagnosed in nearly 30% of patients harboring systemic solid tumors, mainly cancers of lung, breast and skin, but also renal cell carcinoma and gastrointestinal cancer. They typically occur at the grey-white matter junction in the brain hemispheres (75%), followed by cerebellum (21%) and brainstem (4%) [29].

Their management requires a multidisciplinary approach to select the most appropriate treatment for each patient. Treatment choices, according to prognostic classification, include surgery, radiation therapy, systemic chemotherapy, targeted therapy and immunotherapy administered in an isolated or combined manner, with the aim of achieving symptom control and increased survival [27,29,30].

In this setting, the relevant role of surgery in the management of BM in terms of progression free-survival (PFS) and overall survival (OS), as well as improvement in quality of life, has been attested since the 1990s [31,32]. Today, surgery is recommended for carefully selected patients [3,8] and offers several advantages such as immediate relief of symptoms from mass effect, treatment of intracranial hypertension and histological sample to confirm or establish a diagnosis [27].

Two main resection techniques are commonly adopted, each carrying its own pros and cons: “en-bloc” resection which involves tumor removal in a single piece and “piecemeal” resection which involves excision in several small pieces. One or the other should be selected according to patient and pathology features.

The “en-bloc” resection technique, first described by Salvati et al. in 1996 [11], involves removing the entire tumor as a single piece. It is performed by first obtaining the tumor subpial circumferential isolation through an initial corticectomy which is then followed by brain tumor dissection proceeding through deeper zones of brain parenchyma all around the tumor until reaching the bottom of the lesion. This method follows the principles of oncological resection decreasing the tumor cells spillage and minimizing blood loss, which bring a decreased risk of leptomeningeal dissemination [13], postoperative complications and recurrence rates. In addition, it improves hemostasis, resulting in shorter operative time and less complications for highly vascularized metastases [14]. “Piecemeal” resection involves “getting into the tumor” and removing it in small pieces. It is obtained via internal debulking until regions of normal cortex and white matter appear. The main advantage of this technique is to preserve healthy brain and related neurological functions from injury. On the other hand, this approach may result in troublesome bleeding during resection which may reduce visualization and discrimination between vessels feeding the tumor and normal pial arteries and veins, making hemostasis more difficult and increasing the risk of iatrogenic ischemic injuries. During “en-bloc” resection, cortical landmarks are most readily identified prior to the start tumor resection which is performed at the gross boundaries of the lesion. Conversely, during the “stay within the tumor” technique, working from inside the tumor toward the functional cortex, the surgeon must decide when to stop the resection, balancing the risk of leaving residual tumor with the risk of causing iatrogenic injury to healthy brain tissue. As a corollary technique, “en-bloc” provides larger intact specimens for histopathology and research purposes, which results in more accurate diagnosis. Numerous studies attested the superiority of the “en-bloc” over the “piecemeal” technique of BM resection in terms of patients’ outcomes [12,13,15]. Therefore, en-bloc should be the first choice when surgery is indicated in BM; nevertheless, the selection of which surgical technique to adopt depends either on patient-related factors, such as the their clinical condition and functional neuroanatomy but also on pathology related factors such as the histopathology of the primary disease. From the microscopic point of view, most BM are well demarcated, with variable perivascular growth (so-called “vascular cooption”) and/or a more diffuse infiltration (“pseudogliomatous” growth) in the adjacent brain parenchyma [33]; typically, they are circumscribed by a rim of gliotic tissue that separates the tumor from the surrounding healthy brain tissue which provides a safe dissection plane of resection.

We would like to underline that many authors suggest the role of the “no internal touch technique” to prevent the risk of iatrogenic leptomeningeal metastatic disease [13,34]. For the same reason, we suggest to avoid the use of an ultrasonic aspirator that seems to play a crucial role in tumoral cells spillage [5] (Table 2).

## 5. Conclusions

En-bloc resection of BM, if feasible, is the gold standard technique because it reduces the rate of complications, the risk of local recurrence and the risk of LMD. It is suitable especially for more superficial lesions located in non-eloquent areas. This technique should be used especially in presence of BM located in the posterior fossa or near the CSF pathway. Moreover, the en-bloc technique should be primarily chosen to surgically treat BM from breast cancer and melanoma.

## Figures and Tables

**Table 1:** Summary of main findings on brain metastases according to the resection technique from the literature review. PFS: Progression-Free Survival; OS: Overall Survival; EBR: En-bloc Resection; PR: Piecemeal Resection; LMD: Leptomeningeal Disease; S: Surgery; SRS: Stereotactic Radio Surgery.

Authors/ Year	Nature of Study/Number of Patients	Parameter Investigated	Results
Salvati et al. 1996 [11]	Retrospective Surgical series/ 19 pts	OS	EBR associated to better OS (median 8 months)
Suki et al. 2008 [12]	Retrospective Surgical series/260 pts	LMD	PR (137 cases) associated to higher risk of LMD than EBR (123 cases; RR 3.4, 95% CI 1.43–8.12, *p* = 0.006)
Suki et al. 2009 [13]	Retrospective Surgical series/542 pts	LMD	PR (191 cases) associated to higher risk of LMD than EBR (hazard ratio for PR, 5.8; 95% confidence interval, 1.9–17.2; *p* = 0.002; EBR-351 patients-hazard ratio 2.7; 95% confidence interval, 1.3–5.6; *p* = 0.009)
Patel et al. 2010 [14]	Retrospective Surgical series/570 pts	Local Recurrence	PR (201 patients) associated to higher risk of local recurrence than EBR (369 patients) with a hazard ratio of 1.7, 95% CI 1.1–2.6, *p* = 0.03
Hassaneen et al. 2010 [15]	Retrospective Surgical series/ 29 pts	OS	EBR associated to better OS (median of 11.7 month) in a multivariate analysis (EBR vs. PR; *p* = 0.008)
Patel et al. 2015 [16]	Retrospective Surgical series/ 1033 pts (62% EBR; 38% PR)	Complications rate	EBR associated to lesser complication rates (13% for patients undergoing EBR and 19% for patients undergoing PR; *p* = 0.007)
Ahn et al. 2012 [5]	Retrospective Surgical series/ 242 pts	LMD	39 patients (16%) developed LMD at a median of 6.0 months PR associated to higher risk of LMD than EBR group (HR 4.08)
Atalar et al. 2013 [17]	Retrospective Surgical series/ 165 pts	S + SRS and LMD	median OS of 17 months 21/165 patients (13%) developed LMD at a median of 5 months No differences according to the resection technique
Johnson et al. 2016 [18]	Retrospective Surgical series/ 465 pts	S + SRS vs. S and LMD	12% of patients experienced LMD at a median of 6.0 months No differences according to the resection technique
Press et al. 2019 [19]	Retrospective Surgical series/ 134 pts	Hemorrhagic and cystic BM and LMD	22.4% of patients experienced LMD at a median of 24 months No relevance of resection technique at multivariate analysis
Demaerel et al. 2019 [20]	Retrospective Surgical series/ 52 pts	Outcome	EBR (87% of cases of supratentorial and in 50% of infratentorial lesions) associated to minor risk of LMD
Bruzzaniti et al. 2022 [21]	Retrospective Surgical series/ 88 pts	Postoperative perilesional edema	EBR gold standard of treatment for BM < 2 cm with high-grade edema (*p* < 0.004 for eloquent areas BM and *p* < 0.001 for non-eloquent areas BM)
Byun et al. 2022 [22]	Retrospective Surgical series/ 197 pts	Recurrence	OS at 2 years was 43% EBR was associated to lower local/distant recurrence rate (1-year PFS: 79% vs. 62%, *p* = 0.02)
Tewarie et al. 2021 [23]	Meta-analysis/2105 pts	LMD	The median incidence of LMD was 16.1% with a median time to LMD from BM diagnosis of 6 months (3.8–14 months). PR associated to higher risk of LMD
Kalyvas et al. 2023 [24]	Retrospective Surgical series/ 144 pts	Pachymeningeal failure	PR associated to higher risk of pachymeningeal failure (HR 2.38, *p* = 0.027)

**Table 2:** Main indications, pros and cons of en-bloc and piecemeal resection techniques for brain metastases.

	En-Bloc Resection	Piecemeal Resection
Main Indications	More Superficial lesions Non-eloquent areas Lesions Size < 4 cm	Deeper lesions Eloquent areas Lesions Size > 4 cm Lesions far from CSF pathway
Pro	Lesser risk of leptomeningeal dissemination Lesser risk of recurrences Lesser risk of peri and postoperative complications Lesser intraoperative bleeding Preserving of normal anatomy	Lesser healthy brain manipulation Easier tumor removal
Cons	greater need for microsurgical skills necessity to minimally push and transgress healthy brain	Higher risk of leptomeningeal dissemination Higher risk of recurrences Higher risk of peri and postoperative complications Higher intraoperative bleeding

## Data Availability

No new data were created or analyzed in this study.

## Supplementary Materials

The following supporting information can be downloaded at: https://www.mdpi.com/article/10.3390/jpm14111110/s1, Video S1: operative technique.
